# Supplementary figures and images for: Genomic stability of self-inactivating rabies
Source: eLife. 2023 Nov 3;12:e83459. doi: 10.7554/eLife.83459 (PMC10666929; doi:10.7554/eLife.83459)

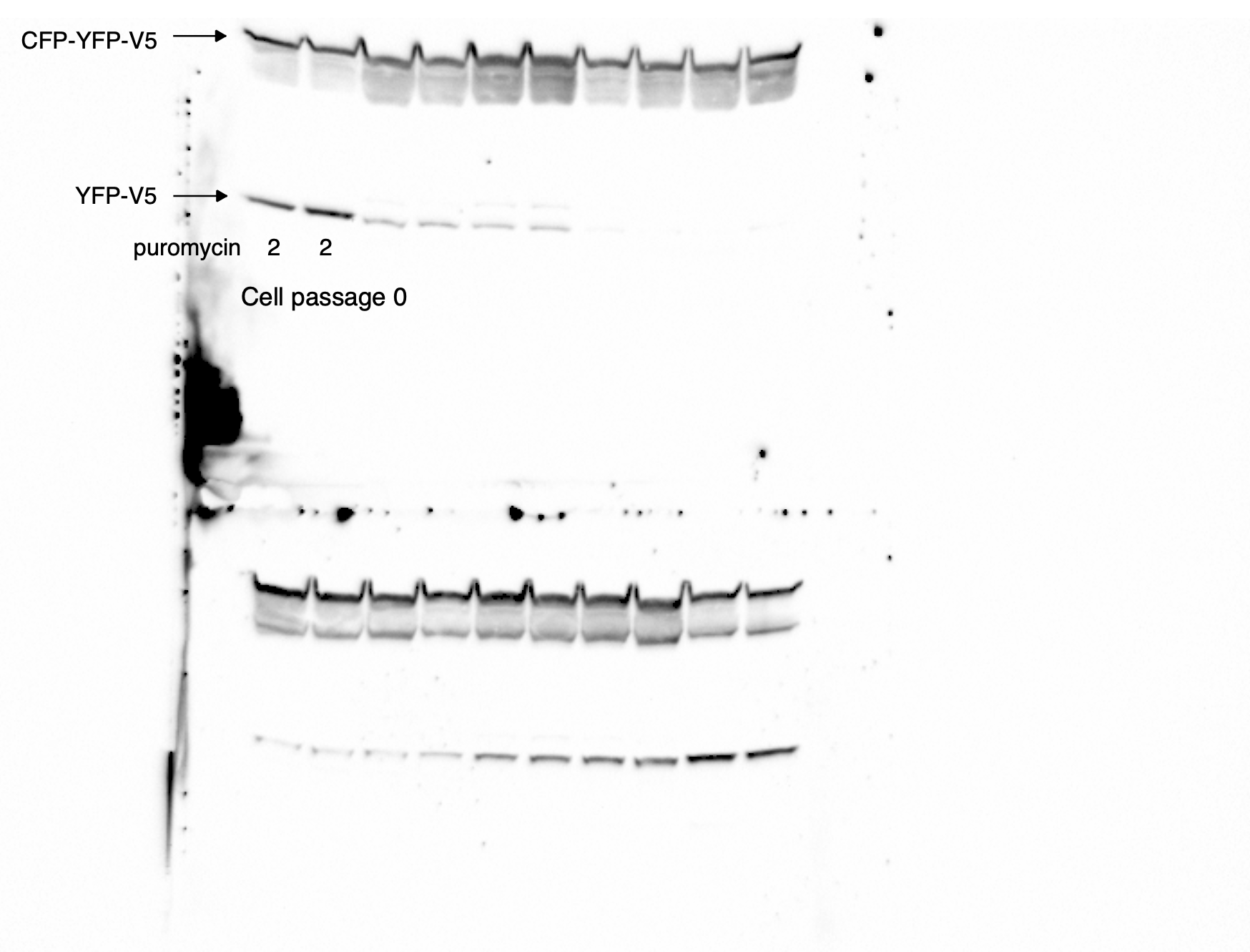

Supplement: Figure 2—source data 1. [file elife-83459-fig2-data1.zip › Figure 2-Source data/Gel 3/Annotated_Gel_TEVp_P0.tif]

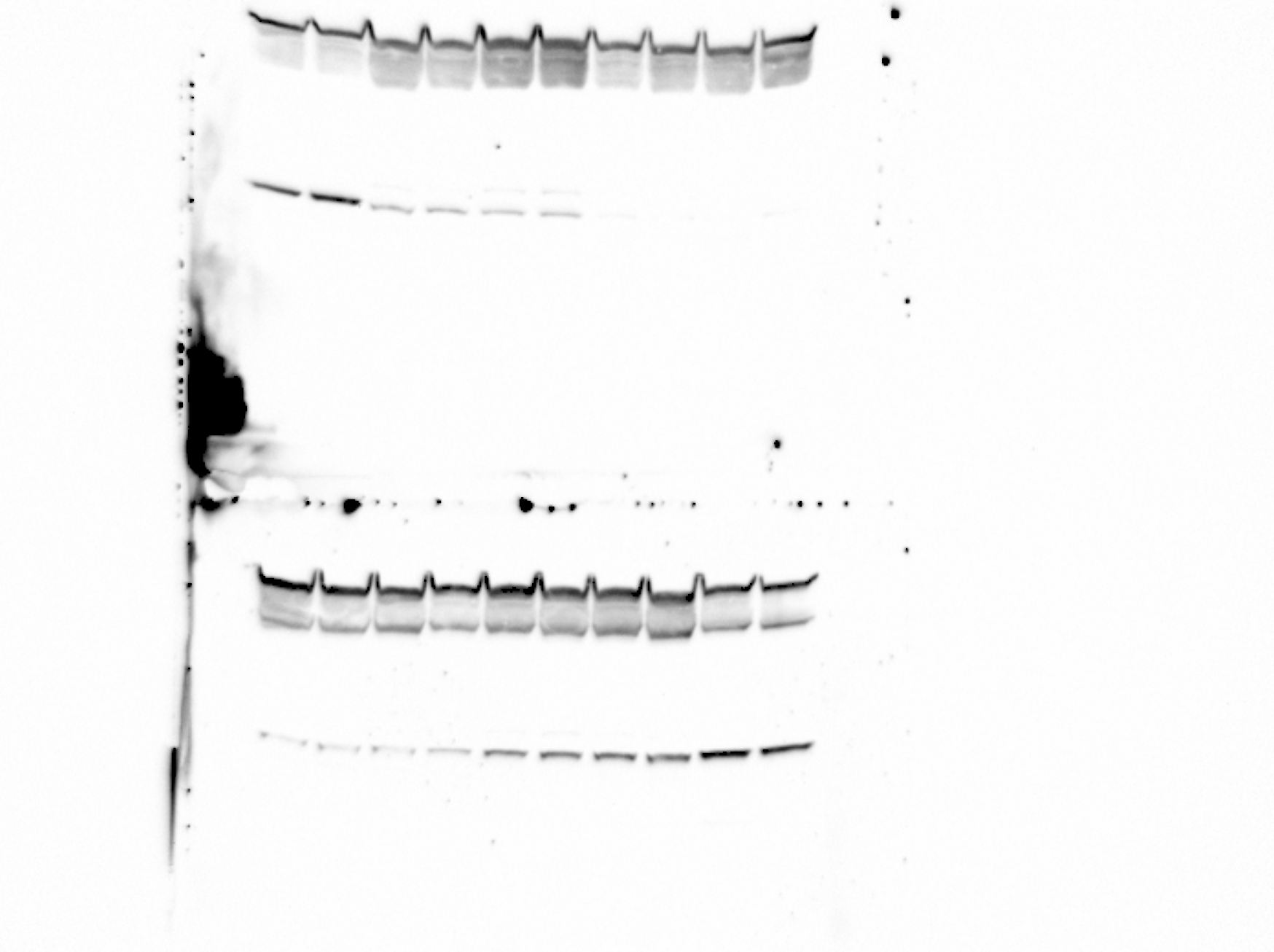

Supplement: Figure 2—source data 1. [file elife-83459-fig2-data1.zip › Figure 2-Source data/Gel 3/Gel_TEVp_P0.tif]

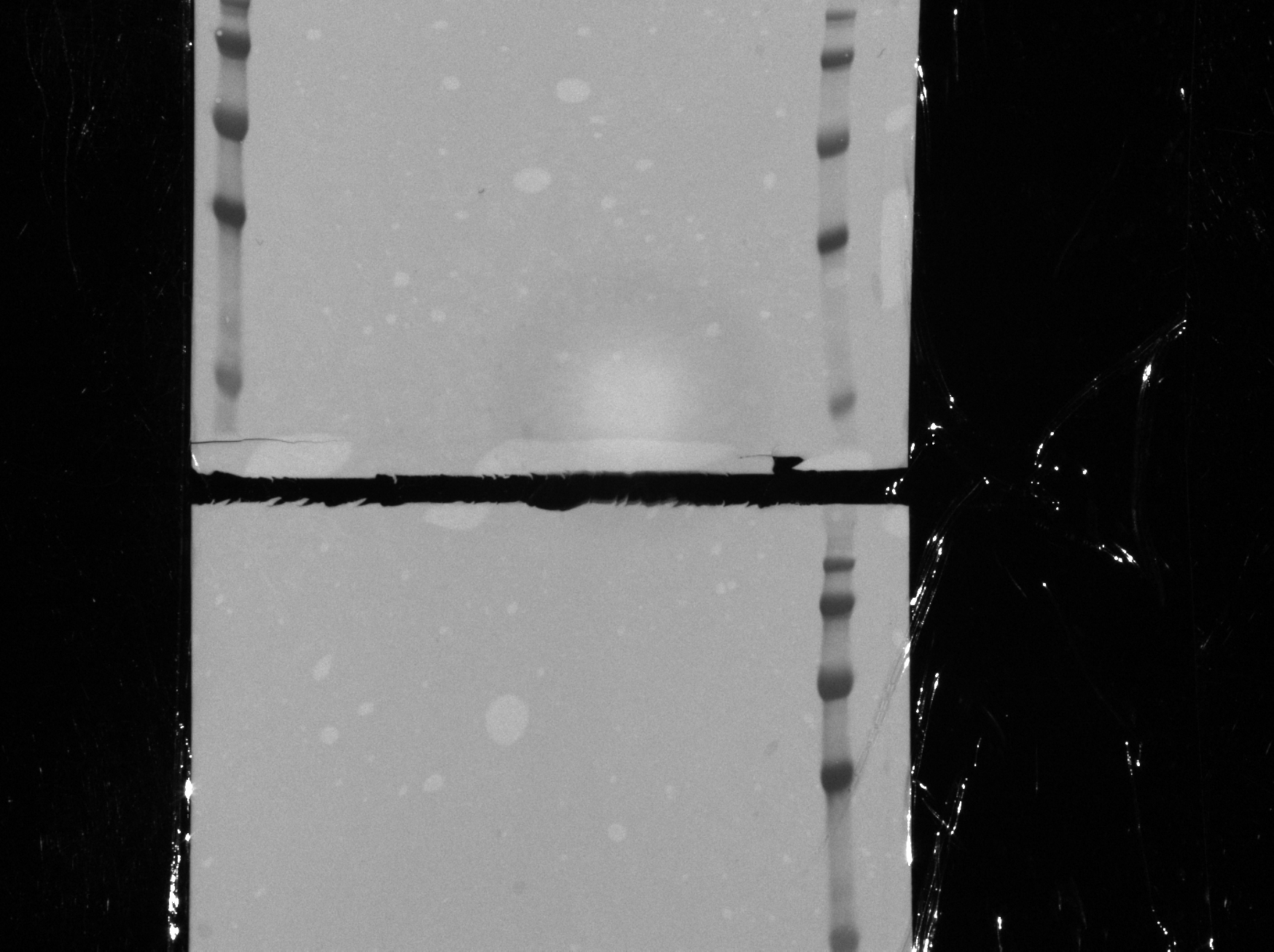

Supplement: Figure 2—source data 1. [file elife-83459-fig2-data1.zip › Figure 2-Source data/Gel 3/Gel_TEVp_P0_Ladder.tif]

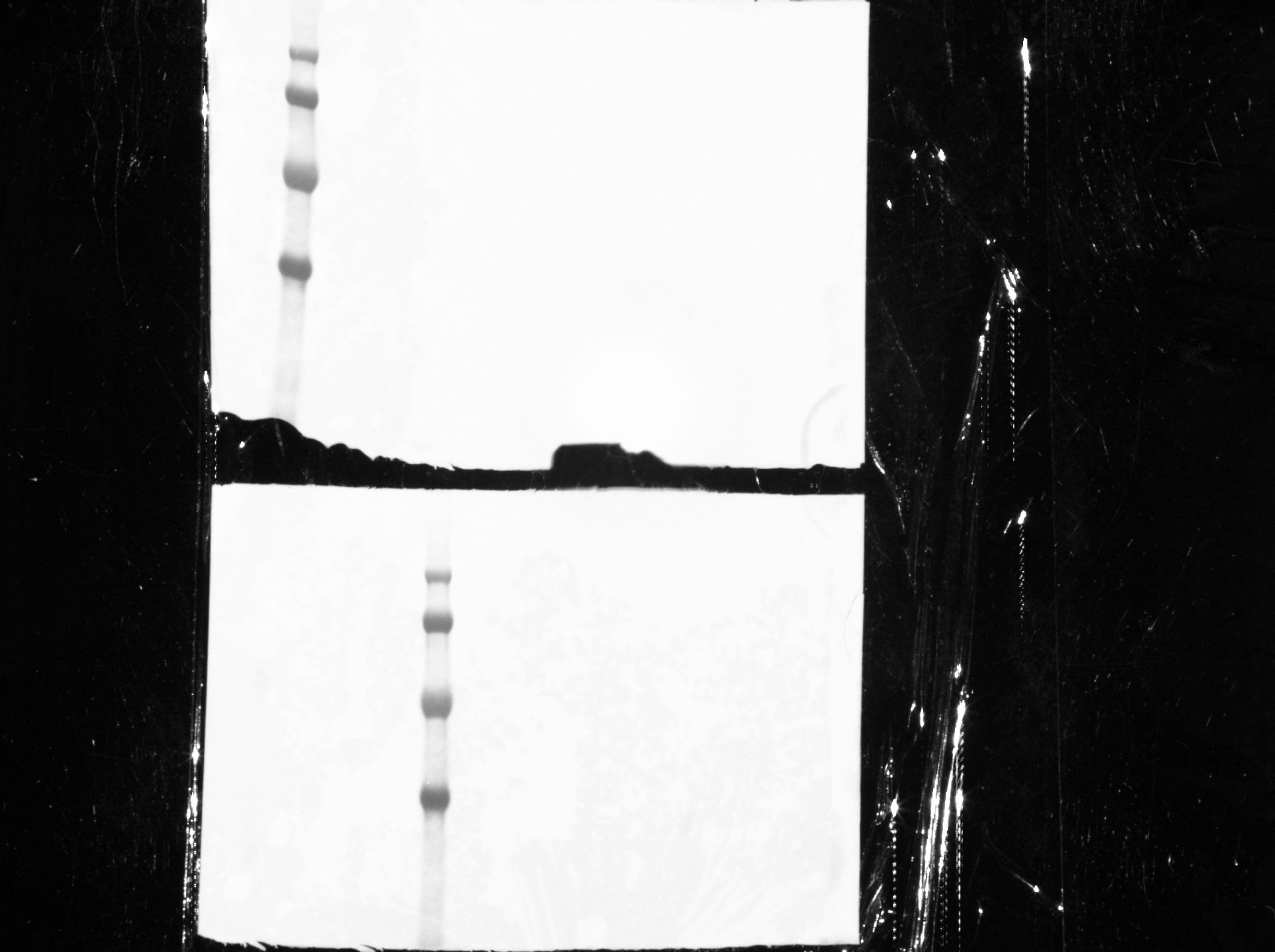

Supplement: Figure 2—source data 1. [file elife-83459-fig2-data1.zip › Figure 2-Source data/Gel 1/Gel_TEVp_P2-4_Ladder.tif]

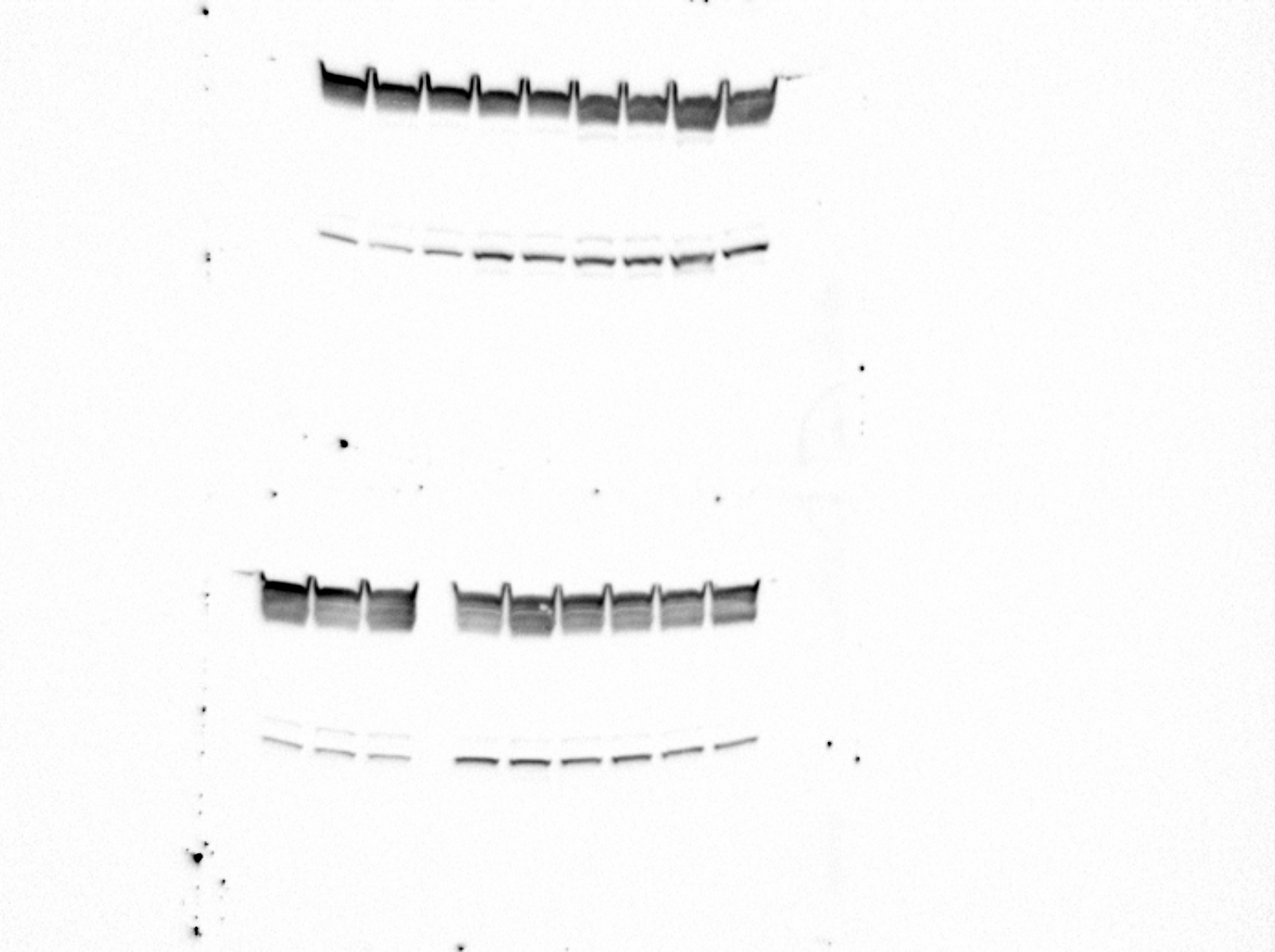

Supplement: Figure 2—source data 1. [file elife-83459-fig2-data1.zip › Figure 2-Source data/Gel 1/Gel_TEVp_P2-4.tif]

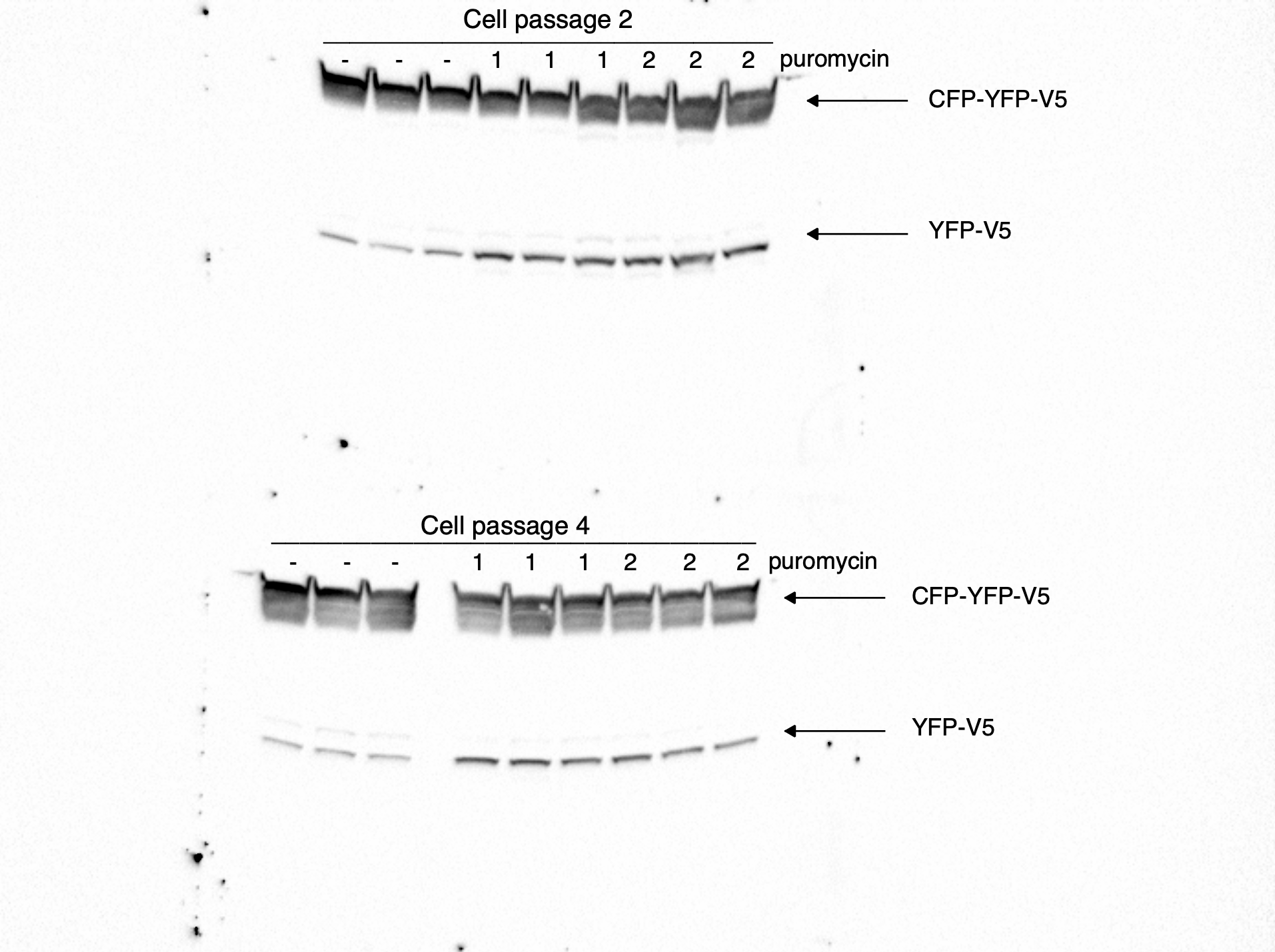

Supplement: Figure 2—source data 1. [file elife-83459-fig2-data1.zip › Figure 2-Source data/Gel 1/Annotated_Gel_TEVp_P2-4.tif]

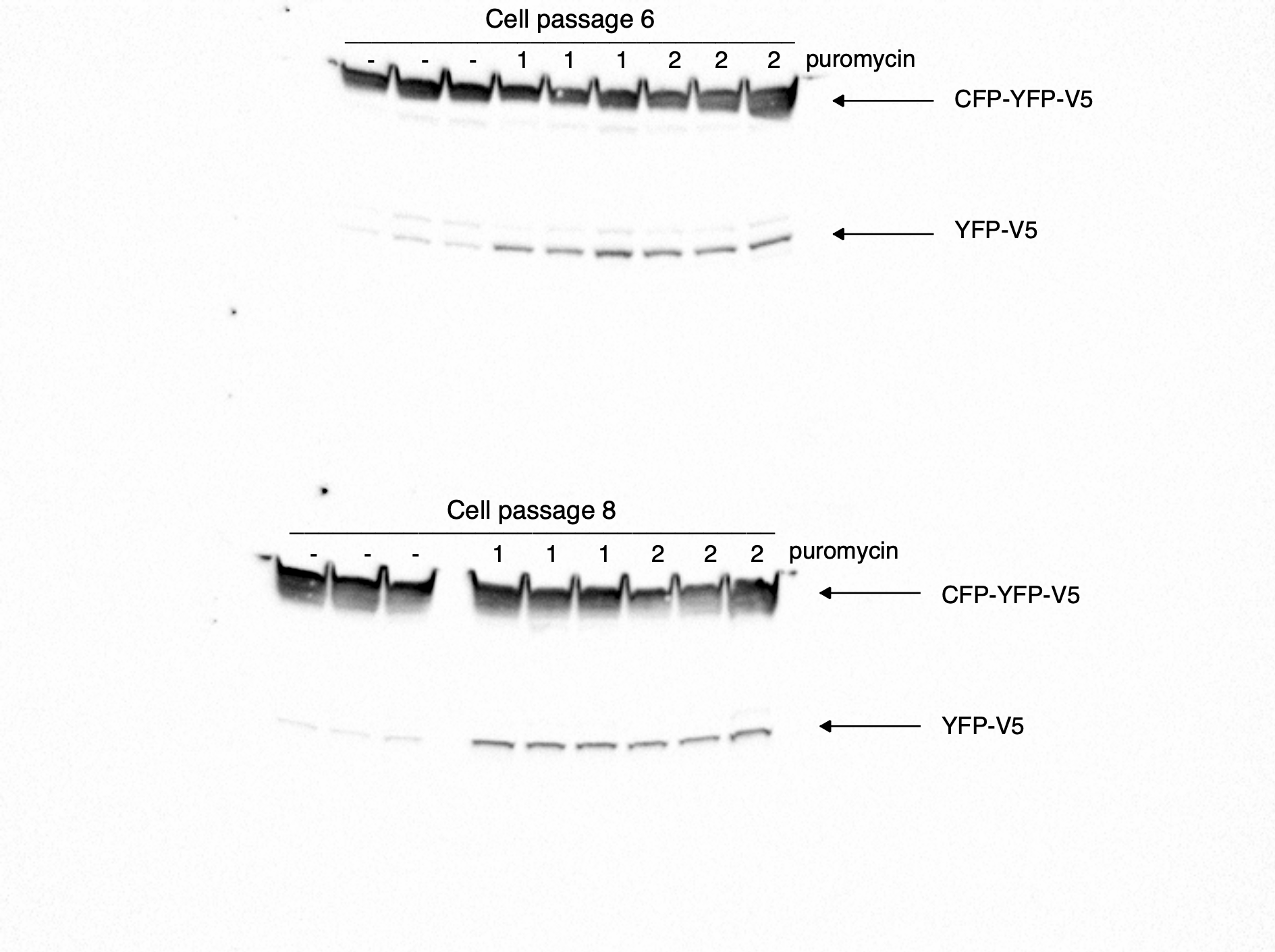

Supplement: Figure 2—source data 1. [file elife-83459-fig2-data1.zip › Figure 2-Source data/Gel 2/Annotated_Gel_TEVp_P6-8.tif]

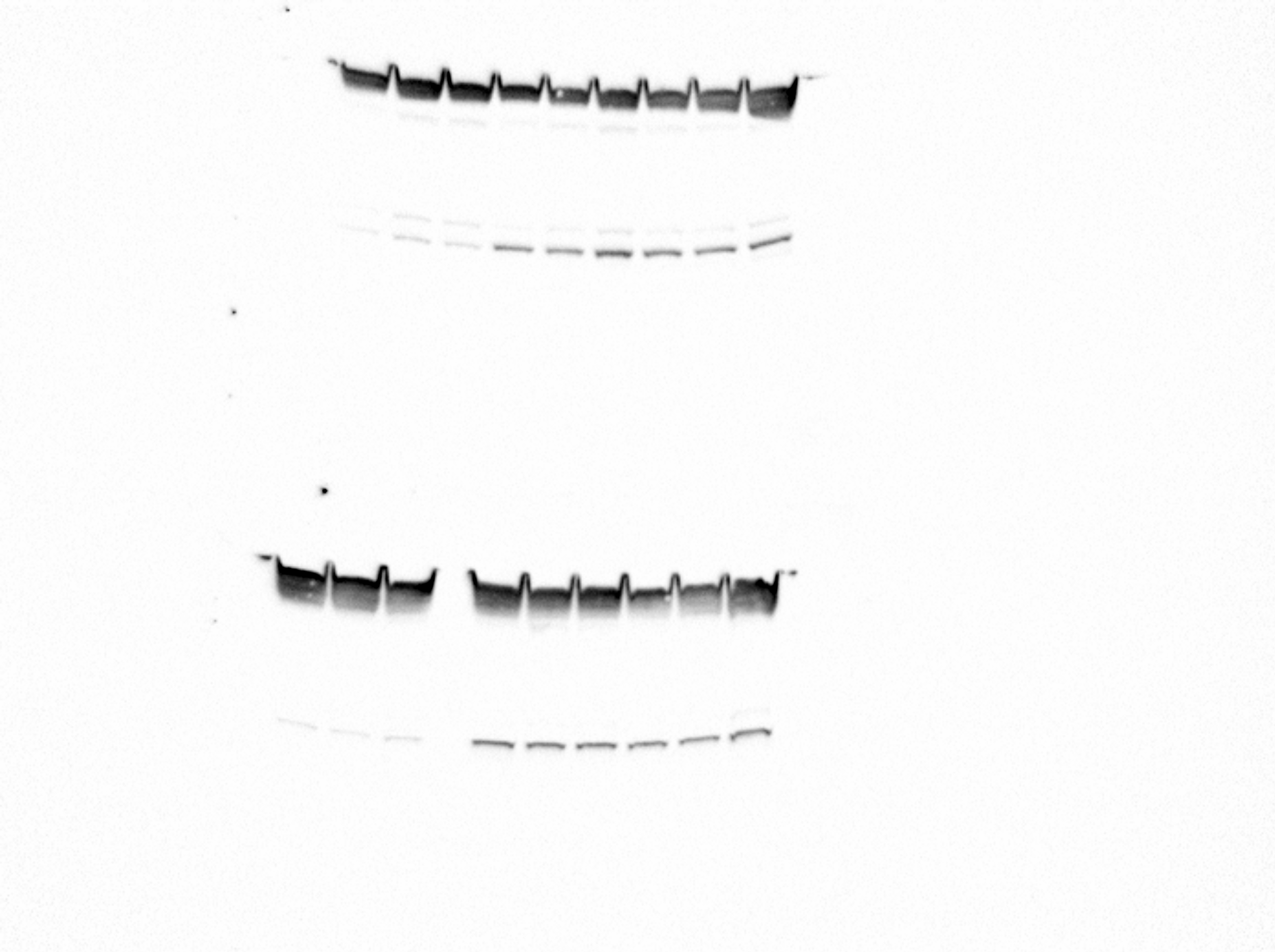

Supplement: Figure 2—source data 1. [file elife-83459-fig2-data1.zip › Figure 2-Source data/Gel 2/Gel_TEVp_P6-8.tif]

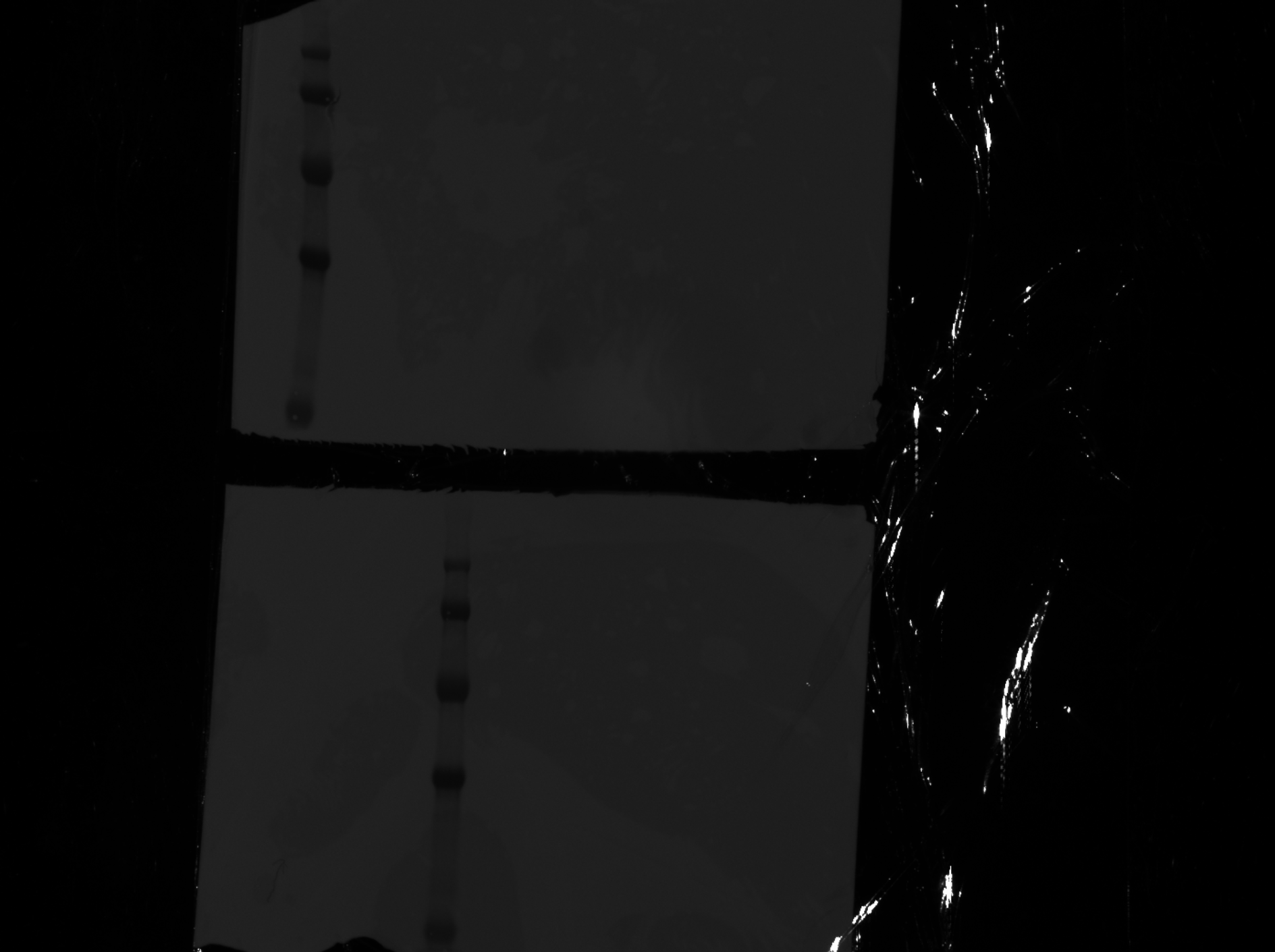

Supplement: Figure 2—source data 1. [file elife-83459-fig2-data1.zip › Figure 2-Source data/Gel 2/Gel_TEVp_P6-8_Ladder.tif]
